# OMICS Applications for Medicinal Plants in Gastrointestinal Cancers: Current Advancements and Future Perspectives

**DOI:** 10.3389/fphar.2022.842203

**Published:** 2022-02-04

**Authors:** Rongchen Dai, Mengfan Liu, Xincheng Xiang, Yang Li, Zhichao Xi, Hongxi Xu

**Affiliations:** ^1^ School of Pharmacy, Shanghai University of Traditional Chinese Medicine, Shanghai, China; ^2^ Engineering Research Center of Shanghai Colleges for TCM New Drug Discovery, Shanghai, China; ^3^ Shuguang Hospital, Shanghai University of Traditional Chinese Medicine, Shanghai, China

**Keywords:** gastrointestinal cancers, omics, medicinal plants, multi-omics, precision medicine, tumour heterogeneity

## Abstract

Gastrointestinal cancers refer to a group of deadly malignancies of the gastrointestinal tract and organs of the digestive system. Over the past decades, considerable amounts of medicinal plants have exhibited potent anticancer effects on different types of gastrointestinal cancers. OMICS, systems biology approaches covering genomics, transcriptomics, proteomics and metabolomics, are broadly applied to comprehensively reflect the molecular profiles in mechanistic studies of medicinal plants. Single- and multi-OMICS approaches facilitate the unravelling of signalling interaction networks and key molecular targets of medicinal plants with anti-gastrointestinal cancer potential. Hence, this review summarizes the applications of various OMICS and advanced bioinformatics approaches in examining therapeutic targets, signalling pathways, and the tumour microenvironment in response to anticancer medicinal plants. Advances and prospects in this field are also discussed.

## Introduction

Gastrointestinal cancers (GI cancers), mainly colorectal, gastric, hepatocellular, pancreatic and oesophageal cancers, contribute to high cancer-related mortality worldwide with significant morbidity and poor prognosis (Kuntz et al., 2021). Combining early detection and several therapeutic approaches, such as endoscopic therapy, surgery and chemotherapy, could improve the survival rate in patients with GI cancer. However, the therapeutic outcomes depend on numerous factors, including the cancer stage at diagnosis, different cancer subtypes, and patient susceptibility (Abdul-Latif et al., 2020). Unfortunately, current anticancer drug development for GI cancer is still forceless and costly; unavoidable adverse events or drug resistance are also nonnegligible obstacles.

Natural products provide a huge reservoir of components with potential pharmaceutical benefits, including anticancer, neuroprotective, and cardiovascular protection. Despite the complex phytochemical compositions of medicinal plants, modern technologies have identified numerous active anticancer ingredients, such as polysaccharides, triterpenes, flavonoids, proteins and amino acids. In the clinical community, several lead compounds derived from medicinal plants have been widely applied as first-line therapeutic agents. For example, paclitaxel isolated from Pacific yew was proven to be a chemotherapy medication used to treat various types of cancer (Mekhail and Markman, 2002). Moreover, GI cancer patients also benefit from medicinal plants as anticancer adjuvants in therapeutic effect enhancement, adverse reaction reduction, immune function improvement and drug resistance elimination (Hu et al., 2016). Nevertheless, the underlying anti-GI cancer mechanisms upon herbal medicine treatment remain obscure, which has spawned further investigations on the global and systemic identification of key target molecules and signalling pathways.

OMICS is a series of approaches that aim to characterize and quantify the pools of biological molecules within organisms and covers numerous pieces of information, including structures, functions, and dynamics. Over the past decades, the integration of bioinformatic analysis with multiple OMICS datasets has allowed for a comprehensive understanding of genetic, proteomic, epigenetic and metabolic processes and thus has been suggested as a powerful and promising tool to uncover biomarkers and therapeutic targets (Nicora et al., 2020). Genomics covers all approaches that use DNA sequences to elucidate molecular profiles. For example, DNA microarray technology (collections of DNA probes arranged on a shared base) is used in specific gene searches or in gene polymorphism and expression analysis on a large scale (Bednár, 2000). Next-generation sequencing describes revolutionized genomic research and enables high-throughput, massively parallel sequencing of either DNA or RNA (Phillips, 2018). Proteins are the functional performers of genes, and the application of proteomics elaborates the identification and quantification of overall proteins present in a cell, tissue or organism. Genomics and transcriptomics expound the identity of proteins in an organism and seek to understand the structures and functions of particular proteins (Aebersold and Mann, 2003). Metabolomics provides comprehensive profiling of small molecule metabolites in different organisms at various life levels. By utilizing NMR and MS-based metabolomics, identified metabolites contribute to new drug discoveries and biomarker prediction (Spratlin et al., 2009).

**FIGURE 1:**
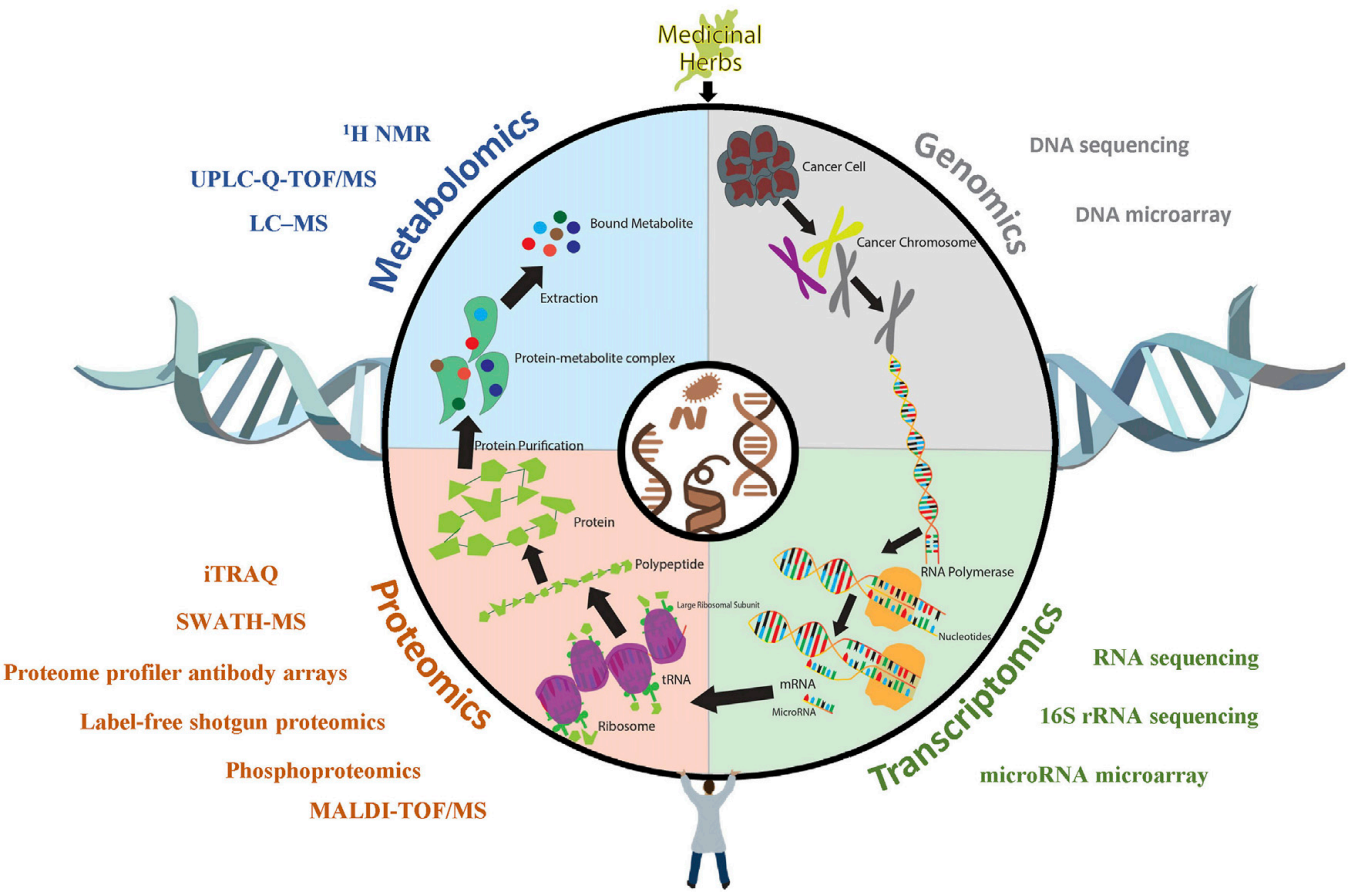
The application of OMICS technologies in anti-GI cancer research. Upon being treated with medicinal herbs, cancer cells undergo a series of biological processes, during which large amounts of molecular alterations are reflected in different dimensions, such as protein and mRNA expression levels and metabolite profiles. Various OMICS technologies, such as sequencing, microarrays, and iTRAQ assays, have been utilized to unravel the respective alterations of cancer in response to medicinal plants.

**FIGURE 2:**
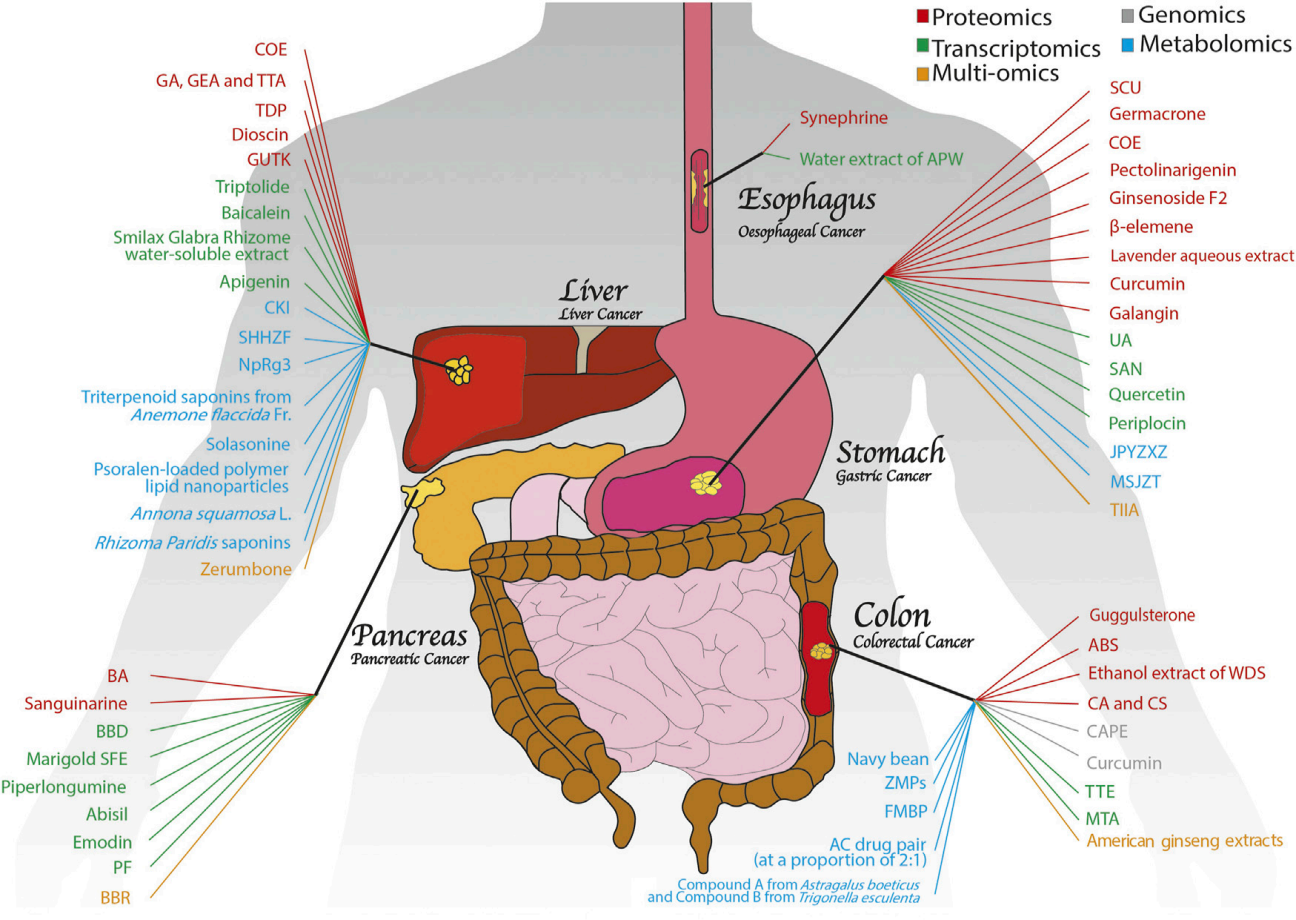
An overview of medicinal plants with anti-GI cancer properties based on OMICS. Numerous medicinal plants have exhibited potent antitumour activities in GI cancers, including colon, pancreatic, gastric, liver and oesophageal cancers. With the help of different OMICS approaches, such as proteomics, genomics, transcriptomics, metabolomics and multi-OMICS, the anti-GI cancer mechanisms of medicinal plants have been explored extensively.

This review focused on the current OMICS technologies utilized for understanding the actions of anticancer medicinal plants in the treatment of GI cancer (Figure 1). We summarized the scientific evidence of OMICS approaches for unravelling the complex signalling interaction networks, key target molecules, tumour microenvironment and host gut microbiota in response to anticancer herbal medicines (Figure 2). Current challenges and opportunities of OMICS were also discussed.

## Applications of OMICS in Gastrointestinal Cancers

### Colorectal Cancer

Colorectal cancer (CRC), one of the most commonly diagnosed gastrointestinal cancers, is characterized by undetectable preneoplastic lesions, rapid deterioration, early metastasis and high mortality (Bray et al., 2018). While early diagnosis and surgical excision combined with chemotherapy have been widely implemented in conquering colorectal cancer, the potential usage of medicinal plants as adjuvants has aroused momentum in this field. The applications of OMICS have merit in identifying anti-CRC active compounds from medicinal plants and helping to unravel underlying anticancer mechanisms, including regulation of apoptosis, anticancer stress, and the host immune response (Table 1).

**TABLE 1:** The applications of OMICS on medicinal plants in Colorectal cancer.

Cancer type	OMICs approach	Active component	Medicinal plant/Formulation	Main anti-tumour mechanism	References
Colorectal cancer	Proteomics	Guggulsterone	*Commiphora mukul* (Hook. ex Stocks) Engl	Induction of intrinsic apoptosis, ↑p53, ↓TRAIL and TNF protein expression, ↓NF-κB signaling pathway	Leo et al. (2019)
		Ankaferd hemostat	*Thymus vulgaris* L.*, Glycyrrhiza glabra* L.*, Vitis vinifera* L.*, Alpinia officinarum* Hance*,* and *Urtica dioica* L	Regulation of glucose metabolism, ↑UCHL1 and RPL5 protein expression	Koçak et al. (2019)
		Ethanol extract of WDS	*Saccharum officinarum* L	↑SELH, ↓phosphorylated NFκB, ↑phosphorylation of SIRT1 and EGFR, ↓phosphorylation of PKA, PKCβ and c-Jun	Bucio-Noble et al. (2018)
		Carnosic acid	*Salvia rosmarinus* Schleid. (syn. *Rosmarinus officinalis* L.)	↑ ER stress	Valdés et al. (2017)
		Carnosol	*Salvia rosmarinus* Schleid. (syn. *Rosmarinus officinalis* L.)	↓20S proteasome catalytic activity	Valdés et al. (2017)
	Transcriptomics	Polyphenol extract	*Thalassia testudinum* Banks & Sol. ex K.D.Koenig	↑ATF4-P53-NFκB gene expression and autophagy stress pathway	Hernández-Balmaseda et al. (2021)
		Methylthioacetic acid	*Cucumis melo* L	Induction of dome formation, ↓ cyclin E2 and CDC25A gene expression	Kamimura et al. (2020)
	Metabolomics	Compound A and Compound B	*Astragalus boeticus* L. and *Trigonella esculenta* Willd	Reversion of drug resistance,↑anti-proliferative activity	Graziani et al. (2018)
		*Astragalus membranaceus*-*Curcuma wenyujin* extract	*Astragalus mongholicus* (Fishch.) Bunge (syn. *Astragalus propinquus* Schischkin) and *Curcuma aromatica* Salisb. (syn. *Curcuma wenyujin* Y.H.Chen and C.Ling)	Regulation of several metabolic alterations	Sun et al. (2021)
		Foxtail millet bran peroxidase	*Setaria italica* (L.) P.Beauv	↓GPL metabolism, ↓PCYT1α and PCYT2 expression	Shan et al. (2020)
		American ginseng extract	*Panax quinquefolius* L	Regulation of metabolites including branched-chain amino acids, organic acids, fatty acids and carbohydrates	Xie et al. (2015)
		Navy bean extract	*Phaseolus vulgaris* L	Regulation of metabolic pathways,↑amino acids, lipids, and bean-derived metabolites	Baxter et al. (2018)
		ZMP	*Ziziphus jujuba* Mill	↑fecal-microbiota diversity and probiotics (*Bifidobacterium*, *Bacteroides*, *Lactobacillus* and *Clostridium_sp_K4410MGS-306*), ↑the concentration of SCFAs	Ji et al. (2019)
	Genomics	Caffeic acid phenethyl ester or Kaempferol	N.A.	Abolishment of DNA mutations, such as PIK3CA, KIT and ABL1	Budisan et al. (2019)
		Curcumin	*Curcuma longa* L	Reversion of CAC-induced DNA CpG methylation downregulation	Guo et al. (2018)
	Multi-omics (Transcriptomics and metabolomics)	American ginseng extract	*Panax quinquefolius* L	↑ glutamine and linolenic acid metabolites, ↓ proinflammatory cytokines, ↑beneficial intestinal microbiome populations (*Firmicutes*), ↓harmful ones (*Bacteroidales* and *Verrucomicrobia*)	Wang et al. (2016)

“↑” represents increase, promote or up-regulate, while “↓” represents inhibit, suppress, decrease or down-regulate, NA, represents not available.

#### Proteomics

Leo et al. (2019) demonstrated that guggulsterone (GS), a plant sterol extracted from the gum resin of the tree *Commiphora mukul* (Hook. ex Stocks) Engl., significantly blocked HCT116 cell proliferation. Incorporating a mass spectrometry-based label-free shotgun proteomics approach and proteome profiler antibody arrays characterized novel proteomic signatures and identified significant protein expression differences between GS-treated and untreated HCT116 cells, including p53, TRIAL, TNF-α/NF-kB, and apoptosis-related proteins. Differentially expressed proteins evaluated by proteomics indicated that Ankaferd hemostat (ABS), a mixture containing plant extracts of *Thymus vulgaris* L. *Glycyrrhiza glabra* L.*, Vitis vinifera* L.*, Alpinia officinarum* Hance, and *Urtica dioica* L., affected cellular and metabolic processes, such as glucose, fatty acid and protein metabolism, in Caco-2 colon cancer cells. Moreover, ABS induced alterations of several cancer targets and upregulated tumour suppressor proteins (UCHL1 and RPL5) (Koçak et al., 2019)*.* SWATH-MS-based proteomics identified that the anti-inflammatory response caused by the ethanol extract of whole dried sugarcane (WDS) influenced the NFκB pathway by upregulating the oxidative stress regulator SELH and reducing phosphorylated NFκB. Further phosphoproteomics studies indicated that WDS promoted the phosphorylation of the cell stress regulators SIRT1 and EGFR while impeding the phosphorylation of PKA, PKCβ and c-Jun in an LPS-stimulated inflammatory model of SW480 colon cancer cells (Bucio-Noble et al., 2018). Moreover, proteomic approaches can help discriminate different mechanisms of action between compounds with similar structures. Carnosic acid (CA) and carnosol (CS) are two orthodiphenolic diterpenes extracted from *Salvia rosmarinus* Schleid*.* (syn. *Rosmarinus officinalis* L.). They share an abietane carbon skeleton with hydroxyl groups at positions C-11 and C-12, while CS has a lactone moiety across the B ring, and CA has a free carboxylic acid group. A proteomics study based on dimethyl labelling combined with nano-LC–MS/MS revealed that the cellular response to CA induced ER stress, and CS treatment directly inhibited 20S proteasome catalytic activity in HT-29 cells, suggesting that these two structurally similar bioactive compounds induced distinct anticancer mechanisms against CRC (Valdés et al., 2017).

#### Transcriptomics

*Thalassia testudinum* Banks & Sol. ex K.D.Koenig polyphenol extract (TTE) suppressed angiogenesis and cancer cell proliferation in SW480 cells and a syngeneic CT26 allograft murine model. Transcriptome profiling and Ingenuity Pathway Analysis showed that TTE exerted anti-CRC activity by promoting autophagic stress and ATF4-P53-NFκB signalling (Hernández-Balmaseda et al., 2021). Administration of methylthioacetic acid (MTA) extracted from *Cucumis melo* L. facilitated the formation of multicellular and cystic structures (termed domes) in human CRC RCM-1 cells, which is a promising differentiation therapy that aims to alleviate cancer malignancy without massacring normal cells. A microarray demonstrated that MTA-induced dome formation is at least partially associated with the downregulation of cyclin E2 and CDC25A, two crucial genes involved in G1 phase cell cycle control (Kamimura et al., 2020).

#### Metabolomics

Chronic inflammation caused by inflammatory bowel disease (IBD), including ulcerative colitis and Crohn’s disease, plays a critical role in tumorigenesis of the gastrointestinal tract (Lichtenstern et al., 2020). Mechanistically, DNA hypermethylation, reactive oxygen species (ROS) production and gut microbiota imbalance facilitate IBD, subsequently leading to the initiation and progression of colitis-associated carcinogenesis (CAC) (Rogler, 2014; Gagnière et al., 2016). A rapid ^1^H NMR-based metabolomic approach identified 31 primary metabolites present in mixtures of fourteen Fabaceae species of Mediterranean vegetation that showed cytotoxicity against colon cancer cells, among which Compound A from *Astragalus boeticus* L. and Compound B from *Trigonella esculenta* Willd. were the two strongest components (Graziani et al., 2018). The *Astragalus membranaceus* (Fishch.) Bunge (syn. *Astragalus propinquus* Schischkin)-*Curcuma aromatica* Salisb. (syn. *Curcuma wenyujin* Y.H.Chen and C. Ling) (AC) drug pair (at a proportion of 2:1) demonstrated significant inhibitory effects on orthotopic transplantation colon tumour growth and metastasis in CT-26 tumour-bearing mice. Intriguingly, a UPLC-Q-TOF/MS metabolomics approach showed that AC treatment reversed several metabolic alterations induced by colon cancer, among which all-trans retinoic acid, a clinical anticancer chemotherapy drug, was significantly upregulated (Sun et al., 2021). One of the hallmarks of classical CAC is the metabolic imbalance resulting from abnormally elevated glycerophospholipids (GPLs). Shan and colleagues analysed the serum metabolite profiles of an AOM/DSS-induced CAC mouse model after foxtail millet bran peroxidase (FMBP) treatment by utilizing untargeted LC–MS-based metabolomics. The results speculated that FMBP blocked GPL metabolism by reducing the expression levels of key metabolic enzymes involved in phosphatidylcholine and phosphatidylethanolamine (such as PCYT1α and PCYT2), resulting in insufficient adenosine triphosphate to maintain CRC growth (Shan et al., 2020).

Plant-based dietary intake confers multiple beneficial bioactive components to the host and prevents humans from developing various diseases; in contrast, a high-fat diet will induce more adenomas and carcinomas (Baltgalvis et al., 2009). American ginseng attenuated Western-style high-fat diet-induced metabolic perturbation, reduced gut inflammation and tumorigenesis, and ameliorated the lifespan of Apc^Min/+^ mice. Mechanistically, the metabonomic profiles showed that American ginseng markedly altered several metabolites, including branched-chain amino acids, organic acids, fatty acids and carbohydrates (Xie et al., 2015). Evidence suggests that fibre intake exhibits a negative correlation with the overall mortality of CRC (Song et al., 2018). In a randomized controlled trial, sufficient fibre intake by ingestion of dietary navy bean (35 g/day) for 4 weeks modulated the stool metabolome of overweight and obese CRC survivors compared with the vehicle control. Using a nontargeted metabolomics approach, navy bean consumption increased the abundance of amino acids, lipids, and bean-derived phytochemicals and protected against CRC by altering major metabolic pathways such as sterol, lysine, fatty acid, amino and inositol metabolism (Baxter et al., 2018).

Emerging evidence has highlighted the crucial role of the gut microbiota in potentiating intestinal carcinogenesis, and medicinal herbs can regulate gut dysbiosis by triggering the immune response, modulating microbial composition and activating immunological signalling pathways (Wong et al., 2017). Short-chain fatty acids (SCFAs) are produced when nondigestible plant carbohydrates reach the proximal colon and are metabolized by various microbiota, which alleviate CRC by modifying the gut microbial and immune systems (Gniechwitz et al., 2007; Tian et al., 2018). *Ziziphus jujuba* Mill. polysaccharides (ZMPs) modulated gut microbial communities by alleviating AOM/DSS-reduced fecal microbiota diversity and promoting the abundance of probiotics, including *Bifidobacterium*, *Bacteroides*, *Lactobacillus* and *Clostridium* sp*._K4410MGS-306,* in a C57BL/6 mouse model (Ji et al., 2019). Comparing the fecal metabolomics profiles with the model group, dietary ZMP consumption reduced intestinal pH in CRC mice and markedly increased the concentration of SCFAs (Ji et al., 2019).

#### Genomics

Applications of genomics can help identify the precise genes regulated by medicinal drugs. Next-generation sequencing revealed that either caffeic acid phenethyl ester (CAPE) or kaempferol abolished CRC-induced pathogenic DNA mutations, such as PIK3CA, KIT and ABL1, in RKO and HCT-116 cells (Budisan et al., 2019). By utilizing single-base-resolution DNA methylation sequencing, Guo et al. (2018) revealed that curcumin, the yellow pigment isolated from the rhizomes of turmeric (*Curcuma longa* L.), reversed CAC-induced downregulation of DNA CpG methylation in azoxymethane (AOM)- and dextran sodium sulfate (DSS)-induced CAC C57BL/6 mouse models.

#### Multi-OMICS

Oral administration of American ginseng (*Panax quinquefolius* L*.*) attenuated AOM/DSS-induced colitis and colon carcinogenesis in the A/J mouse model. Serum and fecal metabolomics analysis demonstrated that American ginseng reversed the AOM/DSS-induced decreases in glutamine and linolenic acid, these two metabolites exhibited anticancer potential by protecting the gut mucosa and suppressing the release of proinflammatory cytokines. Moreover, 16S rRNA sequencing revealed that American ginseng extracts maintained a healthy enteric microbiome community and protected against pathologic processes by obviously increasing beneficial intestinal microbiome populations such as *Firmicutes* and decreasing harmful populations such as *Bacteroidales* and *Verrucomicrobia* (Wang et al., 2016).

### Gastric Cancer

Gastric cancer (GC) initiates with chronic gastritis and progresses to gastric atrophy, intestinal metaplasia, dysplasia, and finally to adenocarcinoma (Tan and Fielding, 2006). By utilizing various OMICS approaches, the unravelled anti-GC mechanisms upon medicinal herb treatment have been largely extended, including but not limited to mediating key molecules involved in apoptosis, cell cycle arrest, DNA repair, and cytoskeleton reorganization (Table 2).

**TABLE 2:** The applications of OMICS on medicinal plants in gastric cancer.

Cancer type	OMICs approach	Active component	Medicinal plant/Formulation	Main anti-tumour mechanism	References
Gastric cancer	Proteomics	Scutellarein	*Scutellaria baicalensis* Georgi	Induction of apoptosis, ↓PIK3CB and CIP2A protein expression	Saralamma et al. (2020)
		Germacrone	*Curcuma zedoaria* (Christm.) Roscoe	Induction of cell cycle arrest, apoptosis and autophagosome formation, ↓ HBXIP protein expression	Fang et al. (2020)
		Ethyl acetate extract	*Celastrus orbiculatus* Thunb	↓ HSP27 protein expression, ↓NF-κB/Snail pathway	Zhu et al. (2014)
		Pectolinarigenin	Rutaceae Juss. family (*Citrus* L. genus)	↓DDX4,↑ LRSAM1 protein expression	Lee et al. (2018)
		Ginsenoside F_2_	*Panax ginseng* C.A.Mey	↑RPL26, ↓PRR5, RPS15, CISD2, Bcl-xl and NLRX1 protein expression	Mao et al. (2016)
		β-elemene	*Curcuma aromatica* Salisb. (syn. *Curcuma wenyujin* Y.H.Chen and C.Ling)	Induction of apoptosis, ↓TOPIIα, ↑PAK1IP1 and BTF protein expression	Liu et al. (2014)
		Aqueous extract	*Plectranthus ecklonii* Benth	↓Annexin1, anolase1 and HSP70 protein expression	Zamanian-Azodi et al. (2012)
		Curcumin	*Curcuma longa* L	Induction of apoptosis	Cai et al. (2013)
		Galangin	*Alpinia officinarum* Hance	Induction of apoptosis, ↑Uch-L1, ↓GSTP protein expression	Kim et al. (2012)
	Transcriptomics	Ursolic acid	Various plants, such as *Prunella vulgaris* L	Regulation of Hippo pathway,↑RASSF1, ↓YAP1, FOXM1, KRAS and BATF genes	Kim et al. (2019)
		Sanguinarine	*Sanguinaria canadensis* L	↓miR-96–5p, miR-29c-3p and MAP4K4 mRNA expression, ↑MAPK/JNK signaling pathway	Dong et al. (2019)
		Quercetin	Various plants, such as *Asparagus africanus* Lam	Induction of apoptosis, ↑ROS production, ↓mitochondrial membrane potential, ↑TP53INP1, ↓VEGFB and CDK10 gene expression	Shang et al. (2018)
		Periplocin	*Periploca sepium* Bunge	Induction of apoptosis, ↑ERK1/2-EGR1 pathway	Li et al. (2016)
		Ethanol extract	*Smilax glabra* Roxb	Induction of intrinsic apoptosis	Gao et al. (2011)
	Metabolomics	Water extract	Jianpi Yangzheng Xiaozheng	↑L-glutamine, L-leucine, L-alloisoleucine, and L-valine, ↓gluconolactone metabolism	Hou et al. (2021)
		MSJZT extract	Modified Si Jun Zi Tang	Regulation of amino acid, glycolysis, and lipid metabolism, ↓LDH, GS and PCYT2 mRNA and protein expression	Nie et al. (2019)
	Multi-omics (Transcriptomics and proteomics)	Tanshinone IIA	*Salvia miltiorrhiza* Bunge	↓glucose consumption and pyruvate production, induction of apoptosis and DNA damage,↑p53, ↓AKT protein expression, ↑PSMB3, ↓RS2 protein expression	Lin et al. (2015)

“↑” represents increase, promote or up-regulate, while “↓” represents inhibit, suppress, decrease or down-regulate.

#### Proteomics

Scutellarein (SCU), a flavone that is abundantly present in *Scutellaria baicalensis* Georgi, promotes cell death by inducing apoptosis in GC AGS and SNU484 cells. To verify the altered proteins after SCU treatment in GC cells, a comparative proteomics technique that integrated two-dimensional gel electrophoresis (2-DE), mass spectrometry (MS) and MALDI-TOF/MS analysis was implemented. The expression levels of two oncogenic molecules, phosphatidylinositol 4,5-bisphosphate 3-kinase catalytic subunit β isoform (PIK3CB) and protein phosphatase 2A (CIP2A), were significantly downregulated upon SCU treatment. Molecular docking studies suggested that SCU could directly bind to PIK3CB with ideal affinity (Saralamma et al., 2020). Germacrone, a monocyclic sesquiterpene isolated from *Curcuma zedoaria* (Christm.) Roscoe, inhibited GC by inducing cell cycle arrest at the G0/G1 phase, inducing apoptosis and promoting autophagosome formation. Label-free proteomic and bioinformatic analyses revealed that germacrone significantly downregulated the protein level of hepatitis B X-interacting protein (HBXIP), which is aberrantly highly expressed in GC patient tumour tissues. Furthermore, overexpressing HBXIP effectively alleviated germacrone-induced cell cycle arrest and apoptosis, whereas si-HBXIP enhanced the inhibitory effect of germacrone on GC cell proliferation, suggesting that HBXIP was a potential target of germacrone in treating GC (Fang et al., 2020). Overexpression of HSP27, the most downregulated protein in response to *Celastrus orbiculatus* Thunb. ethyl acetate extract (COE) identified by proteomics, significantly abrogated the inhibitory effect of COE on SGC-7901 cells. Mechanistically, overexpressing HSP27 alleviated COE-inhibited phosphorylation of IκBα and nuclear translocation of NF-κB p65 and Snail (Zhu et al., 2014). In addition, proteomic approaches provided essential information for understanding the anti-GC mechanisms of pectolinarigenin present in citrus fruits (Lee et al., 2018), Ginsenoside F2 extracted from ginseng (Mao et al., 2016), β-elemene extracted from *Curcuma aromatica* Salisb. (syn. *Curcuma wenyujin* Y.H.Chen and C.Ling) (Liu et al., 2014), Lavender aqueous extract (Zamanian-Azodi et al., 2012), Curcumin isolated from *Curcuma longa* L. (Cai et al., 2013), and Galangin (Kim et al., 2012). Numerous identified proteins in response to these medicinal plants were found to be involved in cell cycle arrest, apoptosis, and other processes.

#### Transcriptomics

Ursolic acid (UA), a natural compound extracted from many herbal medicines, inhibited GC proliferation, invasion and migration in SNU484 and SNU638 cells. Microarray assays combined with Gene Ontology analysis further determined that UA markedly upregulated the Hippo pathway upstream target gene Ras association domain family (RASSF1) and downregulated the Hippo pathway downstream target gene YAP1 together with the oncogenes FOXM1, KRAS, and BATF. More importantly, RASSF1 silencing significantly reversed UA-induced upregulation of p-YAP protein expression, suggesting that UA diminished tumorigenesis *via* the Hippo pathway by regulating RASSF1 (Kim et al., 2019). Sanguinarine (SAN), a benzophenanthridine alkaloid extracted from *Sanguinaria canadensis* L., suppressed GC BGC-823 cell proliferation both *in vitro* and *in vivo*. Microarray analysis following PCR verification revealed that SAN abolished the GC-induced abnormal expression of miR-96-5p, miR-29c-3p and MAP4K4 mRNA. These results indicated that SAN exhibited its anti-GC activity by inhibiting these miRNAs and activating the MAPK/JNK signalling pathway (Dong et al., 2019). Quercetin, a common component found in natural plants, induced apoptotic cell death in AGS human gastric cancer cells by augmenting ROS production and decreasing mitochondrial membrane potential (ΔΨm). A cDNA microarray assay further revealed that quercetin-altered differentially expressed genes were involved in the apoptosis pathway, such as TP53INP1, VEGFB and CDK10 (Shang et al., 2018). Similarly, a microarray assay revealed that Periplocin extracted from *Periploca sepium* Bunge and *Smilax glabra* Roxb. (SGR) exerts anticancer effects on gastric cancer cell lines by regulating both intrinsic (mitochondrial) and extrinsic (FAS and TNFR signalling) apoptotic pathways (Gao et al., 2011; Li et al., 2016).

#### Metabolomics

In a clinical trial of 36 GC patients, the traditional Chinese herbal formulation Jianpi Yangzheng Xiaozheng (JPYZXZ) reduced adverse drug reactions and improved the quality of life of patients after chemotherapy. Metabolomic analysis revealed that GC patients undergoing chemotherapy exhibited metabolic deficiencies in L-glutamine, L-leucine, L-alloisoleucine, and L-valine, which were reversed by JPYZXZ treatment. In addition, the clinical anti-GC mechanism of JPYZXZ was related to the inhibition of increased gluconolactone metabolism caused by GC (Hou et al., 2021). Modified Si Jun Zi Tang (MSJZT) is another Chinese herbal formulation that shows anticancer effects in nude mouse GC models. HILIC and UHPLC-Q-TOF/MS-based metabolomics approaches revealed that modified MSJZT decreased the mRNA and protein levels of LDH, glutamine synthetase, and PCYT2 in mouse plasma. These results suggested that the mechanisms underlying the anti-GC effects of MSJZT were at least partially mediated by regulating GC-induced energy metabolism dysfunction, such as amino acid, glycolysis, and lipid metabolism (Nie et al., 2019).

#### Multi-OMICS

Lin et al. integrated next-generation sequencing-based RNA-seq transcriptomics with quantitative proteomics to uncover that tanshinone IIA (TIIA), a diterpene quinone isolated from *Salvia miltiorrhiza* Bunge, inhibited glucose metabolism in GC cells. Mechanistically, TIIA reduced glucose consumption and pyruvate production by affecting the protein expression levels of G6PI, LDHB, MDH1, PCK2 and PGK1. Moreover, increased p53 and decreased AKT expression were found in response to TIIA-induced apoptosis and DNA damage, which may stimulate aerobic glycolysis in GC. Corresponding with the proteomics results, TIIA upregulated the PSMB3 protein level and downregulated the RS2 protein level, which are involved in cell cycle arrest and DNA repair (Lin et al., 2015).

### Liver Cancer

Hepatocellular carcinoma (HCC) is mainly induced by liver cirrhosis due to viral infection or the excessive use of alcohol and exposure to aflatoxin. Anti-vascular mimicry (VM) has become a promising strategy to inhibit HCC progression by destroying the tumour blood supply system. OMICS technologies help characterize the anti-HCC mechanisms of numerous medicinal plants, including the induction of apoptosis, autophagy, and DNA damage and the regulation of several noncoding RNAs.

#### Proteomics

*Celastrus orbiculatus* Thunb. extract (COE) effectively suppressed VM formation in MHCC97-H cells and a tumour xenograft mouse model. Proteomics analysis identified that the expression of EphA2 was the most significantly decreased protein among 103 downregulated proteins upon COE treatment. Clinical resection of tumour tissues with VM structures from HCC patients expressed higher EphA2 than VM structure-free tissues, while blocking EphA2 by COE resulted in decreased cell invasion and damaged VM formation (Chu et al., 2021). A proteomics study revealed that three derivatives from Gamboge, namely, gambogic acid (GA), gambogenic acid (GEA) and 1,3,6,7-tetrahydroxyxanthone (TTA), inhibited HCC cell proliferation and induced apoptosis by regulating stathmin 1 (STMN1) and 14-3-3σ. The following gain- and loss-of-function studies indicated that overexpressing STMN1 decreased the sensitivity of HCC cells to GA and GEA, whereas silencing STMN1 enhanced the sensitivity. Consistently, 14-3-3σ silencing reversed the suppressive effect of TTA on cell growth and apoptosis induction. Interestingly, bioinformatics analysis *via* AutoDock Vina modelling predicted that TTA interacted directly with chain A of 14-3-3σ (Wang et al., 2009; Fu et al., 2012a). Proteomic profiling analysis found that Hsp27 was one of the most significantly downregulated proteins in response to xanthone (TDP) isolated from *Garcinia oblongifolia* Champ. ex Benth. Silencing Hsp27 inhibited cell growth and induced apoptosis through a caspase-dependent mitochondrial pathway in HepG2 cells, whereas enforcing Hsp27 expression rescued these suppressive effects of TDP (Fu et al., 2012b). Dioscin extracted from *Dioscorea oppositifolia* L. inhibited tumour growth in diethylnitrosamine (DEN)-induced primary liver cancer rats and HCC xenograft nude mice by triggering apoptosis, autophagy, and DNA damage. The iTRAQ assay identified that the expression of TP53-inducible glycolysis and apoptosis regulator (TIGAR) was markedly decreased by dioscin, which was further verified by real-time PCR and Western blotting assays *in vitro* and *in vivo*. Knockdown of TIGAR aggravated the inhibitory effects of dioscin by increasing p53 and inhibiting the Akt/mTOR and CDK5/ATM pathways in SMMC7721 cells and a tumour xenograft nude mouse model (Mao et al., 2019). Proteomic analysis revealed that GUTK isolated from the *Garcinia yunnanensis* Hu markedly upregulated actin-binding protein profilin 1 (PFN1) expression. Tissue microarray data showed that a reduction in PFN1 expression was more common in advanced human HCC, which was associated with a low survival rate. Moreover, overexpression of PFN1 mimicked the effects of GUTK on decreasing the expression of tumour metastasis markers, including F-actin and other proteins involved in actin nucleation, branching and polymerization (Shen et al., 2016). The anti-HCC mechanisms of other medicinal plants unravelled using proteomic approaches are listed in Table 3.

**TABLE 3:** The applications of OMICS on medicinal plants in liver cancer.

Cancer type	OMICs approach	Active component	Medicinal plant/Formulation	Main anti-tumour mechanism	References
Liver cancer	Proteomics	*Celastrus orbiculatus* extract	*Celastrus Orbiculatus* Thunb	↓EphA2 expression, inhibition of cell invasion and VM formation	Chu et al. (2021)
		Gambogic acid and gambogenic acid	*Garcinia hanburyi* Hook. f	Induction of apoptosis,↓STMN1 protein expression	Wang et al. (2009)
		1,3,6,7-tetrahydroxyxanthone	*Garcinia oblongifolia* Champ. ex Benth	Induction of apoptosis,↑14-3-3σ protein expression	Fu et al. (2012a)
		TDP	*Garcinia oblongifolia* Champ. ex Benth	Induction of apoptosis, ↓Hsp27 protein expression	Fu et al. (2012b)
		Dioscin	*Dioscorea oppositifolia* L	Induction of apoptosis, autophagy, and DNA damage, ↓TIGAR mRNA and protein expression, ↑p53 protein expression, ↓Akt/mTOR and CDK5/ATM pathways	Mao et al. (2019)
		GUTK	*Garcinia yunnanensis* Hu	↑ PFN1, ↓F-actin protein expression	Shen et al. (2016)
		Polysaccharides	*Viscum coloratum* (Kom.) Nakai	↑Vitronectin, ↓Histone H3.1 and Cytoskeletal 9 mRNA and protein expression	Chai and Zhao, (2017)
		Ethanol extract	*Antrodia Cinnamomea* T.T. Chang and W.N. Chou	Induction of apoptosis and ER stress	Chen et al. (2020)
		PZH extract	Pien Tze Huang	↑IL-6, NF-κB, TNFR1, TNFR2, p53 and FAK pathway, induction of cell cycle arrest	Fan et al. (2020)
	Transcriptomics	Triptolide	*Tripterygium wilfordii* Hook f	↓ miR-17-92 and miR-106b-25 expression, ↑BIM, PTEN, and p21, ↓ c-Myc and ERCC3 protein expression	Li et al. (2018a)
		Baicalein	*Scutellaria baicalensis* Georgi	Induction of ER stress, apoptosis, oxidative stress and p53 signaling pathway,↓HSPA1A and HSPA1B gene expression, ↓HSP70 protein expression	Ma et al. (2019a)
		Water-soluble extract	*Smilax glabra* Roxb	↓ TGF-β1 signaling	She et al. (2015)
		Apigenin	Various plants, such as *Scutellaria barbata* D.Don	↑11 miRNA expression (hsa-miR-24, hsa-miR-6769b-3p, and hsa-miR-6836–3p, etc.), ↓hsa-miR-181a-5p and hsa-miR-148a-3p expression	Wang et al. (2021)
	Metabolomics	Compound Kushen Injection	*Radix sophorae flavescentis* and *Rhizoma smilacis glabrae*	Regulation of glycometabolism and amino acid metabolism,↑pyruvate, ↓glutamate	Gao et al. (2018)
		SHHZF gramule	Shuihonghuazi	↑linoleic acid and oleic acid uptake and utilization, ↑arachidonic acid metabolites, ↑PEMT activity, ↓bile acid metabolism	Bao et al. (2017)
		NpRg3	*Panax ginseng* C.A.Mey	↑free fatty acids, ↓3-indolepropionic acid and urea, regulation of gut microbiota	Ren et al. (2020)
		Triterpenoid Saponins	*Anemone flaccida* F.Schmidt	↑1,3-diaminopropane, lauric acid, 2,4-diaminobutyric acid 2, and ribitol metabolites, modulation of histidine, arginine, proline, beta-alanine, glycine, serine, and threonine metabolism	Han et al. (2019)
		Solasonine	*Solanum melongena* L	Induction of ferroptosis, ↓GPX4 and GSS activity, ↑lipid ROS level	Jin et al. (2020)
		Psoralen-loaded polymer lipid nanoparticles	*Psoralea corylifolia* L	Reversion of drug resistance, ↓retinoic acid and α-linolenic acid	Li et al. (2021)
		Annonaceous acetogenins	*Annona squamosa* L	Regulation of glutathione, arginine, proline and sphingolipid metabolism	Ma et al. (2019b)
		*Rhizoma Paridis* Saponins	*Paris polyphylla* Sm	↓lactate, acetate, N-acetyl amino acid and glutamine signals	Qiu et al. (2016)
	Multi-omics (Transcriptomics and metabolomics)	Zerumbone	*Zingiber zerumbet* (L.) Roscoe ex Sm	↑DIABLO, CASP3, BNIP3L, DEED and PMAIP1, ↓ERCC2 and HELLS gene expression, ↓PI3K/AKT/mTOR and STAT3 signalling pathways, ↓^13^C_6_-glucose consumption and glycolytic intermediate levels	Wani et al. (2018)

“↑” represents increase, promote or up-regulate, while “↓” represents inhibit, suppress, decrease or down-regulate.

#### Transcriptomics

Triptolide, a structurally unique diterpene triepoxide extracted from *Tripterygium wilfordii* Hook F., induced apoptosis in multiple HCC cell lines in a p53-independent manner. A microRNA microarray identified that triptolide significantly downregulated the expression of two oncogenic miRNA clusters (miR-17-92 and miR-106b-25) in HepG2 cells and a xenograft mouse model, which subsequently upregulated the expression levels of their common target genes, including BIM, PTEN, and p21. Knockdown of these two miRNA clusters enhanced the anticancer activity of triptolide, while their overexpression protected HCC cells from triptolide-induced apoptosis. Further mechanistic studies illustrated that triptolide inhibited these two miRNA clusters by repressing the expression of c-Myc by targeting the ERCC3 protein (Li et al., 2018a). Additionally, transcriptomics approaches have gained insight into various genetic alterations underlying the anti-HCC mechanisms of baicalein isolated from *Scutellariae radix* (Ma et al., 2019a), *Smilax glabra* Roxb. water-soluble extract (She et al., 2015) and apigenin (Wang et al., 2021) (Table 3).

#### Metabolomics

Compound Kushen injection (CKI) inhibited the proliferation and migration of SMMC-7721 cells *in vitro* by inhibiting the protein expression of MMP2, MYC and REG1A and increasing CASP3 protein expression. The ^1^H-NMR metabolomics approach validated twenty-two differential metabolites mapped to key pathways of glycometabolism and amino acid metabolism after treatment with CKI. More specifically, CKI significantly increased the content of pyruvate in the medium while decreasing the uptake of HCC-elevated glutamate in the cell, subsequently attenuating metabolic disorders in hepatoma cells (Gao et al., 2018). A metabolomic study of DEN-induced HCC rats characterized that Shuihonghuazi formula (SHHZF) augmented the uptake and utilization of linoleic acid and oleic acid and increased the content of arachidonic acid metabolites, which improved organism immunity. Moreover, SHHZF also increased the activity of phosphatidylethanolamine N-methyltransferase (PEMT), a liver-specific enzyme that negatively regulates cancer cell proliferation, and suppressed the abnormal metabolism of bile acid. SHHZF regulated these metabolites to a normal level, which could partially explain the mechanism of the therapeutic effects of SHHZF on liver cancer (Bao et al., 2017). Ren et al. developed a novel type of nanomedicine, NpRg3, by conjugating Fe@Fe_3_O_4_ nanoparticles with ginsenoside Rg3. NpRg3 significantly decreased DEN-induced liver tumour nodules, delayed HCC-induced ileocecal morphology, prolonged the survival of HCC mice and inhibited lung metastasis by enhancing the immune response. Metabolomics analysis in mouse serum and liver demonstrated that NpRg3 application inhibited HCC progression by increasing free fatty acids, decreasing 3-indolepropionic acid and urea, and remodelling the unbalanced gut microbiota (Ren et al., 2020). Table 3 also summarizes other usages of metabonomics approaches in identifying metabolite changes in response to triterpenoid saponins from *Anemone flaccida* F. Schmidt (Han et al., 2019), Solasonine isolated from *Solanum melongena* L. (Jin et al., 2020), psoralen-loaded polymer lipid nanoparticles (Li et al., 2021), *Annona squamosa* L. (Ma et al., 2019b), and *Rhizoma Paridis* saponins (Qiu et al., 2016).

#### Multi-OMICS

Zerumbone, a sesquiterpene derived from *Zingiber zerumbet* (L.) Roscoe ex Sm., inhibited HCC tumorigenesis by inducing cell cycle arrest and apoptosis *in vitro* and retarded subcutaneous and orthotopic tumour growth and lung metastasis in NSG mice. A microarray together with a human phosphoprotein array determined that zerumbone induced apoptosis in HCC cells by activating proapoptotic genes (DIABLO, CASP3, BNIP3L, DEED and PMAIP1) and inhibiting antiapoptotic gene (ERCC2 and HELLS) expression; the PI3K/AKT/mTOR and STAT3 signalling pathways were also inhibited. NMR-based metabolomics analysis showed significant suppression of ^13^C_6_-glucose consumption and glycolytic intermediate levels (glycerol-3-phosphate and 3-phosphoglycerate) in zerumbone-treated HCC cells. Further mechanistic studies validated that zerumbone deregulates the expression of glycolysis and pentose phosphate pathway genes, which may be an effective approach to suppress hepatocarcinogenesis (Wani et al., 2018).

### Pancreatic Cancer

Pancreatic cancer is becoming one of the deadliest cancers, with the highest mortality worldwide because of its pathogenic latency and the lack of efficient drugs in the clinic. The overall 5-year survival rate of pancreatic cancer is lower than 5% (McGuigan et al., 2018). OMICS, especially transcriptomics, has revealed a series of oncosuppressor gene and protooncogene alterations upon medicinal plant treatment, which has aroused increasing opportunities to find potential biomarkers and eventually concurrent pancreatic cancer (Table 4).

**TABLE 4:** The applications of OMICS on medicinal plants in pancreatic cancer.

Cancer type	OMICs approach	Active component	Medicinal plant/Formulation	Main anti-tumour mechanism	References
Pancreatic cancer	Proteomics	Betulinic acid	Rhamnaceae, Paeoniaceae, Myrtaceae, and Betulaceae families	↑VAPB, BRE and APOA1, ↓TACO1, RNF167, POLRMT, and DDX49 protein expression	Chiu et al. (2021)
		Sanguinarine	Papaveraceae family, *Sanguinaria canadensis*, *Chelidonium majus*, and *Argemone Mexicana*	↑DUSP4 protein expression	Singh et al. (2015)
	Transcriptomics	Babao Dan	Babao Dan	↑MTUS1, PDGFB, SOD3, and UCHL1, ↓CDK15 and MYBL1 gene expression, ↓MAPK cascade and Wnt signalling pathway	Song et al. (2020)
		Marigold SFE	*Calendula officinalis* L	Induction of autophagic cell death,↑BMP8B gene expression	Gómez de Cedrón et al. (2019)
		Piperlongumine	*Piper longum* L	Regulation of oxidative stress and ER stress	Dhillon et al. (2016)
		Abisil	*Abies sibirica* Ledeb	↑GADD45, DUSP, and DDIT gene families	Kudryavtseva et al. (2016)
		Emodin	*Rheum palmatum* L	↓P16, RASSF1A and ppENK methylation	Zhang et al. (2015)
		Paeoniflorin	*Paeonia lactiflora* Pall	↑HTRA3 gene expression	Li et al. (2017)
	Multi-omics (Metabolomics and transcriptomics)	Berberine	Various plants, such as *Phellodendron amurense* Rupr	Regulation of citrate metabolism and transportation, ↓KRAS, ACLY, ACO1, ↑CDKN2A and SLC25A1 gene expression	Liu et al. (2020)

“↑” represents increase, promote or up-regulate, while “↓” represents inhibit, suppress, decrease or down-regulate.

#### Proteomics

Betulinic acid (BA) derived from the Rhamnaceae, Paeoniaceae, Myrtaceae, and Betulaceae families selectively suppressed pancreatic ductal adenocarcinoma cell (PDAC) proliferation and metastasis without affecting normal pancreatic cells. A protein microarray combined with the Kaplan–Meier plotter database identified that BA upregulated VAPB, BRE and APOA1 protein expression, which may contribute to longer survival times in PDAC patients. Interestingly, BA-downregulated proteins (TACO1, RNF167, POLRMT, and DDX49) were associated with mitochondrial respiratory chain complex I activity, which partially explained the anti-pancreatic mechanisms of BA (Chiu et al., 2021). In addition, a large-scale label-free comparative proteomic approach uncovered multiple proteins, including CUL5, IL33, GPS1 and DUSP4, that are responsible for the anti-pancreatic cancer effect of sanguinarine found in plants of Papaveraceae family. Further qRT-PCR and immunoblot analyses validated that DUSP4 was remarkably upregulated following sanguinarine treatment, which is a possible tumor suppressor that negatively regulates ERK, JNK and MAPK (Singh et al., 2015).

#### Transcriptomics

Intragastric administration of a mixed powder of traditional Chinese medicine called Babao Dan (BBD) significantly decreased volumes and weights in PDAC mouse models. High-throughput RNA sequencing identified 638 upregulated genes (including tumour suppressor genes such as MTUS1, PDGFB, SOD3, and UCHL1) and 259 downregulated genes (including oncogenes such as CDK15 and MYBL1) in response to BBD. These altered genes were associated with BBD-inhibited cancer-related pathways (MAPK cascade and Wnt signalling pathway) and BBD-elevated metabolic activities (steroid hormone biosynthesis and adipocyte lipolysis regulation) (Song et al., 2020). Marigold SFE, a supercritical CO_2_ extract from *Calendula officinalis* L., induced autophagic cell death in MiaPaca-2 pancreatic cancer cells by diminishing mitochondrial respiration and aerobic glycolysis. A comparative gene expression microarray identified that marigold SFE treatment upregulated the expression of bone morphogenetic protein-8B (*BMP8B*), which led to energetic catastrophe and subsequently induced autophagic cell death. More importantly, silencing *BMP8B* reversed the inhibition of mitochondrial oxidative phosphorylation caused by marigold SFE and alleviated ER stress and CHOP expression (Gómez de Cedrón et al., 2019). Consistently, RNA sequencing confirmed several transcriptional changes in response to piperlongumine isolated from long peppers in MiaPaca-2 cells, which were associated with oxidative stress and ER stress (Dhillon et al., 2016). Microarray assays also help identify various alterations in tumour suppressor genes and proto-oncogene expression after treatment of medicinal plants, such as Abisil extracted from *Abies sibirica* Ledeb. (Kudryavtseva et al., 2016) and Emodin isolated from *Rheum palmatum* L. (Zhang et al., 2015) (Table 4). For example, the tumour suppressor gene candidate HTRA3 was significantly augmented after treatment with paeoniflorin (PF) extracted from *Paeonia lactiflora* Pall(Li et al., 2017).

#### Multi-OMICS

Berberine (BBR), an isoquinoline alkaloid widely found in Chinese medicinal plants, suppressed the viability and metastasis of pancreatic cancer cells. A precision-targeted metabolome assay demonstrated that BBR significantly disturbed the energy metabolism of pancreatic cancer cells by damaging mitochondrial structure and affecting tricarboxylic acid cycle activity by influencing citrate metabolism and transportation. Further RNA sequencing revealed that BBR blocked the biosynthesis of fatty acids by mediating ACLY, ACO1 and SLC25A1. Moreover, BBR decreased the expression of the oncogene KRAS and increased the expression of the tumour suppressor gene CDKN2A (Liu et al., 2020).

### Oesophageal Cancers

Oesophageal squamous cell carcinoma (ESCC) is the predominant histological subtype and accounts for the highest incidence of oesophageal cancer cases in China (Table 5). Synephrine, a natural phenolic compound isolated from the leaves of citrus trees, inhibited ESCC proliferation, migration and invasion and enhanced the sensitivity of ESCC cells to fluorouracil (5-FU). Quantitative proteomics identified that synephrine exhibited anticancer effects through inactivating AKT and ERK pathways by downregulating the protein expression of Galectin-3, which was further validated by western blot and qRT–PCR in ESCC cells and tumour xenografts in nude mice (Xu et al., 2018). Gene expression microarray analysis revealed that the water extract of *Andrographis paniculata* (Burm.f.) Nees (APW) exhibited anticancer and antimetastatic activities on ESCC cells by regulating WNT, TGF-β, MAPK, and ErbB signalling pathways and ATP-binding cassette transporters (Li et al., 2018b).

**TABLE 5:** The applications of OMICS on medicinal plants in oesophageal cancer.

Cancer type	OMICs approach	Active component	Medicinal plant/Formulation	Main anti-tumour mechanism	References
Oesophageal cancer	Proteomics	Synephrine	Rutaceae Juss. family (*Citrus* L. genus)	↓AKT and ERK signaling pathways, ↓Galectin-3 protein expression	Xu et al. (2018)
	Transcriptomics	Water extract	*Andrographis paniculata* (Burm.f.) Nees	Regulation of WNT, TGF-β, MAPK, and ErbB signalling pathways and ATP-binding cassette transporters	Li et al. (2018b)

“↑” represents increase, promote or up-regulate, while “↓” represents inhibit, suppress, decrease or down-regulate.

## Discussion and Prospects

Plants are a treasure trove for the research and development of new drugs, with approximately one-quarter of the world’s commonly used drugs now being developed from plant ingredients. However, there are still numerous agents that failed to obtain approval under clinical trials due to their unclear active ingredients and mechanisms. Therefore, multiple OMIC approaches have prompted the development of anticancer agents to an unprecedented level, especially those derived from medicinal plants. Over the past decades, advances in OMICS technology have made great efforts to identify molecular and cellular mechanisms contributing to the development and progression of cancer. To further facilitate the research and discovery of anticancer drugs in medicinal plants, we suggest integrating multiple OMICS analyses, introducing OMICS in precision medicine, and applying single-cell multi-OMICS in research on tumour heterogeneity.

### Integration of Multi-OMICS Approaches in Cancer Research

Single OMICS approaches, such as DNA-seq, microarray and iTRAQ, have revealed intricate anti-GI cancer mechanisms of medicinal plants in different dimensions (such as the gene, RNA, and protein levels) (Chakraborty et al., 2018). However, depicting the anticancer pharmacological actions of medicinal plants at a unidirectional level relying on individual OMICS data is insufficient for discovering the whole profiles of mechanisms. To better understand the cancer-host interactions and key molecular targets of natural anticancer compounds from a systematic and holistic perspective, multidimensional approaches are required and have become a vital step in cancer research. For instance, by integrating quantitative proteomics and transcriptomics, both transcriptional and posttranscriptional genes have been identified in TIIA-treated gastric cancer cells, providing comprehensive and detailed insight into mechanistic studies (Lin et al., 2015). Additionally, combining transcriptomic and metabolomic datasets uncovered a strong correlation between energy metabolism and fatty acid biosynthesis-related genes after BBR treatment (Liu et al., 2020). The emergence of multi-OMICS technologies has greatly impacted the cancer research landscape, bridged the gap between the molecular signatures and phenotypic characteristics of cancer and enabled the identification of therapeutic targets and the development of new drugs in medicinal plants.

### Introduction of OMICS in Precision Medicine

The complexity of cancers and diversity of patients have increased the requirements for precise and personalized medical treatments. Precision medicine is defined as custom-made healthcare of individual patient that is tailored on the basis of genetic, phenotypic, or psychosocial characteristics. Precision medicine that addresses cancer is referred to as “precision oncology,” which seeks to determine tumour-driving networks and to design a personalized therapy in particular patient’s tumour. Numerous OMICS technologies have provided essential information for precision oncology in diagnosing patients, predicting risk, and assessing whether specific treatments are suitable for certain cancer patients. Starting from the field of genomics, next-generation whole-exome and whole-genome sequencing data have largely facilitated the identification of cancer-specific mutations, analysis of chromosomal rearrangements and copy number variations in individual tumours. Recent clinical applications of genome-wide association studies are focusing on drug discovery and repositioning, cancer prognostication, and risk modelling (Sud et al., 2017). Epigenomics merits decimating tumour cells from normal cells and differences within tumour cells during disease progression and drug treatment. For example, the DNA methylation status of the MGMT promoter is currently utilized to determine whether therapeutic agents are effective in glioblastoma treatment (Hegi et al., 2005). Additionally, a majority of metabolomics data have focused on the analysis of plasma or serum samples from patients during diagnosis, which could identify tumour-specific biomarkers without invasive tumour biopsy (Olivier et al., 2019). In particular, some putative metabolic biomarkers have been identified for early-stage detection of colorectal cancer, such as unsaturated free fatty acids (Zhang et al., 2016). Several studies have utilized proteomic approaches to identify cancer-specific biomarkers; however, their promotion of clinical benefit is limited because protein function is regulated by a myriad of mechanisms (Swiatly et al., 2018; Yanovich et al., 2018).

However, it is worth noting that the occurrence of cancer does not necessarily exhibit genetic alterations, nor is it always reflected in metabolite profiles. Therefore, false-negative results from OMICS may lead to the missed diagnosis of cancers, while false-positive results, especially those previously reported pathogenic markers that were actually from benign tissues, may lead to misdiagnosis (Andreasen et al., 2013). For example, nonsynonymous *BARD1* gene variants were previously reported to be associated with increased breast cancer risk. However, later studies found that three previously reported pathogenic variants are benign polymorphisms in the Australian population, indicating that *BARD1* is not accurate enough to represent the high-penetrance susceptibility gene of breast cancer (Gorringe et al., 2008). To avoid these mistakes, large, diverse, and standardized datasets should be established to provide comprehensive profiles of cancer patients. DECIPHER, the first web-accessible database that linked disease phenotype with pathogenic sequence and numbers of variants, was established in 2004 (Swaminathan et al., 2012). The Cancer Genome Atlas (TCGA) characterizes detailed OMICS and clinical data on approximately 85,000 cases of 36 kinds of cancer types (Shah et al., 2016). CancerLinQ, a quality measurement and reporting system raised by the American Society of Clinical Oncology (ASCO), was established to collect bioinformation from large numbers of patients with cancer (Shah et al., 2016). The significance of these standardized datasets not only improves individual patient treatment but also contributes to the discovery of disease and drug mechanisms for the medical community (Schilsky et al., 2014).

### Application of Single-Cell Multi-OMICS in Research of Tumour Heterogeneity

Tumour heterogeneity, including intertumoural heterogeneity (heterogeneity between different tumour types) and intratumoural heterogeneity (heterogeneity within individual tumours), plays essential roles in tumour progression, metastasis and chemoradiotherapy resistance. Previously, high-throughput sequencing techniques have been employed to uncover heterogeneity by profiling genomes, transcriptomes, proteomes and epigenomes of the cellular components. For example, an integrated genome-wide DNA copy number array and a gene expression array were used to classify distinct glioblastoma subtypes of patient tumour tissues (Sottoriva et al., 2013). Exome sequencing transcriptomics was performed to detect both good and poor prognosis gene expression signatures in different regions of the whole primary renal carcinoma (Gerlinger et al., 2012). However, the limitations of these techniques on investigating tumour heterogeneity are obvious since these procedures usually detect the average signals of mixed cell populations rather than individual cells. In this case, single-cell sequencing technologies exhibit great advantages in profiling comprehensive molecular features of tumour tissues (Hoppe et al., 2014).

Single-cell multi-OMICS technologies could characterize hybrid states of cancer cells within a tumour. Using single-cell RNA sequencing (scRNA-seq), different kinds of cells with the potential to regulate the tumour microenvironment have been identified in various cancers, such as epithelial/mesenchymal cells, immune cells and endothelial cells (Pan and Jia, 2021). In addition, single-cell techniques can distinguish cancer cell subpopulations with different biological characteristics. For instance, scRNA-seq revealed several cancer cell subpopulations with differential proliferative and migratory potentials in PDAC, indicating the high intertumour heterogeneity of PDAC (Peng et al., 2019). Quiescent cancer cells (QCCs), a batch of cancer cells among heterogeneous cancer cell populations, temporarily reside in the G_0_/G_1_ phase and are naturally refractory to chemotherapeutic drugs that depend on proliferative mechanisms (Nik Nabil et al., 2021). In addition, QCCs represent a clinically asymptomatic form of dormancy that have the ability to revive from an inert state to rapidly grow, eventually leading to cancer recurrence. As a result, isolation and functional study of QCCs by single-cell multi-OMICS techniques will be a powerful tool to develop therapeutic strategies targeting dormant cancer cells to prevent recurrence (Davis et al., 2019). Studies have discovered active compounds with cytotoxicity against QCCs from herbal medicine (such as GUTK isolated from the *Garcinia genus* and safranal from *Crocus sativus* L.) (Xi et al., 2016; Jiang et al., 2020). Drug resistance is another consequence of tumour heterogeneity, and great progress has been made by utilizing multiple single-cell sequencing methods to uncover drug resistance mechanisms. For instance, the intratumoural heterogeneity and drug-refractory subclones of breast cancer patients were analysed by single-cell transcriptomic analysis after years of chemotherapy treatment. Interestingly, mesenchymal and growth factor signalling pathways, including the epithelial-mesenchymal transition pathway and receptor tyrosine kinases, were enhanced, while antigen presentation and tumour necrosis factor alpha (TNF-α) signalling were reduced (Brady et al., 2018).

In conclusion, the development of high-throughput sequencing technologies has brought unprecedented opportunities in the field of OMICS. With the optimization and maturity of various technologies, the application of multi-OMICS will become more extensive, and it will play a vital role in advancing the progress of anticancer drug research in medicinal plants.
